# Metformin intake associates with better cognitive function in patients with Huntington's disease

**DOI:** 10.1371/journal.pone.0179283

**Published:** 2017-06-20

**Authors:** David Hervás, Victoria Fornés-Ferrer, Ana Pilar Gómez-Escribano, María Dolores Sequedo, Carmen Peiró, José María Millán, Rafael P. Vázquez-Manrique

**Affiliations:** 1 Department of Biostatistics, Health Research Institute La Fe (Hospital Universitario y Politécnico La Fe), Valencia, Spain; 2 Research Group in Molecular, Cellular and Genomic Biomedicine, Health Research Institute La Fe (Hospital Universitario y Politécnico La Fe), Valencia, Spain; 3 CIBER de Enfermedades Raras (CIBERER), Madrid, Spain; 4 Department of Neurology, Hospital Universitario y Politécnico La Fe, Valencia, Spain; Sant Joan de Déu Children's Hospital, SPAIN

## Abstract

Huntington’s disease (HD) is an inherited, dominant neurodegenerative disorder caused by an abnormal expansion of CAG triplets in the huntingtin gene (*htt*). Despite extensive efforts to modify the progression of HD thus far only symptomatic treatment is available. Recent work suggests that treating invertebrate and mice HD models with metformin, a well-known AMPK activator which is used worldwide to treat type 2-diabetes, reduces mutant huntingtin from cells and alleviates many of the phenotypes associated to HD. Herein we report statistical analyses of a sample population of participants in the Enroll-HD database, a world-wide observational study on HD, to assess the effect of metformin intake in HD patients respect to cognitive status using linear models. This cross-sectional study shows for the first time that the use of metformin associates with better cognitive function in HD patients.

## Introduction

Huntington disease (HD, OMIM entry #143100) is a dominant, inherited neurodegenerative disease caused by an abnormal CAG expansion within the first exon of the huntingtin gene, *htt*. This gene encodes a cytosolic protein, huntingtin (Htt) whose function is still unclear, although several roles have been suggested [1]. The CAG expansions found in HD patients encode poly-glutamine tracts (polyQ) which consist of 36 or more Gln residues conferring abnormal toxic properties on the huntingtin protein. Such mutant Htt (mHtt) tends to aggregate both with itself and with other proteins thus perturbing both its own function and the function of the other molecules [2]. Whether mHtt aggregation is cause or consequence of the pathology is a matter of debate [3]. In any case, the resulting cellular function impairment has a particular devastating effect in neurons resulting in their degeneration and cell death. Neuronal loss causes chorea, which is one of the hallmarks of HD along with psychiatric phenotypes and cognitive deterioration. The later can be identified in HD patients many years prior to clinical diagnosis of motor-based changes [4,5,6,7].

Adenosine monophosphate-activated protein kinase (AMPK) has shown potential as an HD druggable target as its activation induces improved neuronal survival [8,9] and mHtt clearance [8] in *in vitro* and *in vivo* models of HD. In some cases a neuroprotective effect has been observed when AMPK activation is induced prior to the occurrence of substantial functional alterations and cell death [8]. This is the case of models of early-stage HD such as *Caenorhabditis elegans* expressing polyQs [10] and striatal neurons from Hdh111Q mice [11]. Metformin is an antiglycemic drug commonly used for the treatment of type-2 diabetes that has a well described AMPK activator effect, although it also has other targets [12,13]. Experiments in several systems suggest that metformin might have a beneficial effect on HD models. In a *C*. *elegans* model in which worms express toxic polyQs in neurons, the presence of metformin reduced neuronal impairment [8]. Furthermore, incubation of striatal cells from Hdh111Q mice in metformin reduced cell death [8]. Finally, in the *in vivo* R6/2 mouse model of HD, male mice treated with metformin had a significantly prolonged survival time [9]. In contrast, over activated forms of AMPK have been found in brain tissue of HD patients together with data from *in vitro* and *in vivo* models [14,15] suggest that AMPK activation during late stages of HD may have deleterious effects, so that AMPK may be best considered as a target to treat HD during early phases of the disease.

Cognitive deterioration can be used as a hallmark of the progression of HD pathology, and hence any observed improvements in cognitive symptoms after a given treatment should reflect some degree of neuronal protection. Based on our previously published data [16], we hypothesized that metformin intake might ameliorate neuronal function, and by extension reduce cognitive decline, in HD patients. A significant number of HD patients, also suffering from type 2 diabetes, have been prescribed metformin. This data is available in the Enroll-HD cohort, which is a worldwide observational study on HD. We therefore explored the existence of an association between metformin intake and cognitive performance in HD individuals by analyzing the Enroll-HD by statistical means. Using linear regression models we have observed an association between metformin use and better cognitive results in different cognitive test, suggesting that metformin may improve cognitive symptoms in HD patients.

## Results

### Clinical characteristics of the sample

Enroll-HD is a world-wide observational study on HD which derives from the integration of two existing HD observational studies: REGISTRY (Europe) and COHORT (North America and Australia). The database includes in-depth clinical records which are collected following finely stablished methods, so the records form different hospitals and countries can be treated homogeneously. The database cohort includes HD patients disease together with pre-manifest carriers of the mutant allele of the *htt* gene (people with 36 or more CAG triplets), and controls. This database also contains detailed information about educational status, drug abuse habits, health conditions (others than HD), medication etc. Regarding HD, Enroll-HD contains a wide range of motor and cognitive tests. We have analyzed on a cohort of Enroll-HD manifest HD patients using linear models, to compare cognitive function between users and non-users of metformin.

Our sample consisted of a set of 7000 individuals from the Enroll-HD cohort (those who had their pharmacologic treatments registered). Of these, 4345 (62.1%) were manifest HD, 1271 (18.2%) were pre-manifest and the remaining 1384 (19.7%) were controls. 7.4% of controls and 2.8% of HD patients were prescribed metformin to treat their type 2 diabetic condition, which is independent of HD. Insulin-dependent diabetics do not regularly use metformin and their pathology has different molecular basis than type 2 diabetes, so they were excluded from this analysis. The proportions of type 2 diabetic individuals among the groups of people in the Enroll-HD database were within the expected range for Western populations [17]. The age profile of the two groups (controls and patients) was similar, although metformin users were 5–10 years older on average. With respect to HD’s status, of those carrying a mutant allele (i.e. >35 CAG triplets) 64.8% (4345) were at the motor-manifest stage (Table 1) and were included in our analysis. The average number of CAG repeats in the carrier population was 43.6 (Table 1). As expected, patients at the motor-manifest stage displayed lower scores in all cognitive tests and also higher values in the UHDRS motor score compared to both carriers at the pre-manifest stage and non-carrier individuals (Table 2). The latter two groups showed similar scores (Table 2). In the control group, the people taking metformin, to treat their type 2 diabetic condition, showed worse cognitive scores than non-metformin users, which suggest that type 2 diabetes has negative consequences on cognitive function in the control population. This is in agreement with previous literature that shows that type 2 diabetes has a deleterious effect over cognitive function (reviewed by Zilliox and coworkers [18]). In contrast, amongst HD patients who were at the motor manifest stage of HD, metformin users, which also were type 2 diabetics, had slightly better cognitive scores than non-metformin users (Table 2).

**Table 1:** Description of the population analyzed from the Enroll-HD database.

	Non-HD individuals (n = 1384)				HD patients (n = 5616)			
	No metformin (n = 1282) (No diabetes)		Metformin (n = 102) (Type 2 diabetes)		No metformin (n = 5456) (No diabetes)		Metformin (n = 160) (Type 2 diabetes)	
	Genotype negative (n = 644)	Family control (n = 638)	Genotype Negative (n = 37)	Family control (n = 65)	Pre- Manifest (n = 1232)	Motor- Manifest (n = 4224)	Pre- Manifest (n = 39)	Motor- Manifest (n = 121)
**Age**	45.6 (14.47) ^a^ 46 (34, 56)	56.09 (11.72) 58 (49, 64.5)	52.97 (12.92) 53 (44, 63)	61.97 (10.38) 63 (55, 69)	41.82 (11.9) 41 (33, 51)	53.32 (12.56) 54 (45, 62)	49.77 (13.05) 50 (38.5, 58)	59.39 (10.92) 59 (52, 68)
**Sex Female Male**	470 (73.0%) 174 (27.0%)	395 (61.9%) 243 (38.1%)	24 (64.9%) 13 (35.1%)	23 (35.4%) 42 (64.6%)	813 (66.0%) 419 (34.0%)	2198 (52.0%) 2026 (48.0%)	26 (66.7%) 13 (33.3%)	58 (47.9%) 63 (52.1%)
**Body Mass Index**	27.67 (6.44) 26.5 (23.4, 30.8)	28.88 (6.16) 28 (24.7, 31.6)	36.05 (8.09) 34.6 (31.6, 39)	34.04 (6.39) 32.7 (29.62, 37.78)	26.8 (5.6) 25.8 (22.75, 29.8)	25.04 (5.1) 24.3 (21.6, 27.58)	33.06 (6.54) 32.4 (28.4, 36.5)	29.5 (6.05) 28.85 (25.1, 32)
**ISCED** ^b^	4.03 (1.14) 4 (3, 5)	3.89 (1.19) 4 (3, 5)	3.27 (1.41) 3 (3, 4)	3.75 (1.24) 4 (3, 5)	3.91 (1.13) 4 (3, 5)	3.37 (1.23) 3 (2, 4)	3.85 (1.11) 4 (3, 5)	3.31 (1.35) 3 (2, 5)
**CAG repeats**	20.22 (3.66) 19 (18, 22)	20.05 (3.41) 19 (17, 22)	20.65 (3.03) 21 (18, 22)	20.15 (3.83) 19 (17, 22)	42.29 (2.72) 42 (41, 43)	44.03 (3.95) 43 (42, 45)	40.87 (2.12) 41 (39.5, 42)	42.34 (2.33) 42 (41, 43)

^a^ Data are presented as mean (SD) and median (1st, 3rd quartile).

^b^ International Standard Classification of Education promoted by UNESCO to standardise.

**Table 2:** Cognitive results among the HD patients and controls ^a^.

	Non-HD individuals			HD patients				
	No metformin (No diabetes)	Metformin (Type 2 diabetes)		No metformin (No diabetes)		Metformin (Type 2 diabetes)		
			Cognitive function change	Pre- manifest	Motor- manifest	Pre- manifest	Motor- manifest	Cognitive function change ^b^
**UHDRS Motorscore**	1.97 (3.32) 1 (0, 3)	2.78 (3.94) 1 (0, 4)	-	3.96 (5.84) 2 (0, 6)	40.99 (22.39) 37 (24, 55)	3.67 (4.59) 3 (0, 5.5)	37.73 (18.7) 36 (25, 48)	-
**Symbol Digit Modality Test**	49.97 (11.55) 51 (44, 57)	42.63 (13.05) 44 (34, 52)	**-14.7%**	48.75 (12.8) 50 (41, 57)	21.82 (13.31) 21 (13, 30)	44.56 (12.5) 45 (36, 52.5)	22.52 (13) 22 (13, 30)	**+3.2%**
**Trail Making Test**	59.27 (34.61) 49 (39, 69)	83.19 (57.14) 63 (47.75, 93.25)	**-40.4%**	61.65 (35.7) 52 (40, 70.75)	156.94 (74.44) 151 (91, 240)	72.21 (56.14) 54 (42.25, 68.5)	157.18 (67.79) 150 (96.5, 240)	**-0.2%**
**Verbal Fluency Test**	21.66 (5.35) 22 (18, 25)	19.59 (6.03) 20 (16, 23)	**-9.6%**	20.94 (5.82) 21 (17, 25)	11.49 (5.91) 11 (7, 15)	19.46 (5.36) 19 (15, 22.5)	12.07 (5.7) 11 (8, 15)	**+5.0%**
**Stroop Interference Test**	42.39 (11.19) 42 (35, 49)	36.08 (9.99) 35.5 (29, 43.25)	**-14.9%**	42.61 (11.27) 43 (35, 50)	22.79 (11.9) 22 (15, 30)	37.18 (11.52) 39.5 (29.25, 46.5)	23.46 (13.73) 22 (15, 29.75)	**+2.9%**
**Stroop Color Naming Test**	74.95 (14.81) 75 (65, 84)	69.96 (14.56) 69.5 (61, 78)	**-6.7%**	71.83 (15.96) 72 (62, 82)	39.93 (19.49) 40 (28, 52)	64.85 (15.81) 67 (53, 77)	40.89 (16.62) 40 (29.75, 50)	**+2.4%**
**Stroop Word Reading Test**	95.04 (18) 96 (85, 105)	86.42 (18.79) 86 (79, 99)	**-9.1%**	91.38 (20.01) 93 (80, 103)	52.58 (24.26) 53 (37, 69)	83.41 (20.99) 85 (74, 96.5)	51.7 (21.33) 50 (37.5, 65)	**-1.7%**
**Cognitive Score**	285.26 (46.69) 285 (255, 314)	258.38 (50.88) 255 (228, 287.5)	**-9.4%**	275.86 (54.09) 279 (241, 312)	158.16 (63.26) 155 (117, 199)	250.55 (58.16) 263.5 (219.3, 289)	158.44 (56.87) 154 (113.25, 191.5)	**+0.2%**

^a^ Controls include *family controls* and *genotype negative* individuals.

^b^ The comparison in performance between HD patients that take metformin and HD patients that do not has been done only with motor-manifest patients.

### HD patients that take metformin show better scores in cognitive tests

In the Enroll-HD study, the assessment of cognitive function used specific neuropsychological tests to assess different domains of cognitive function. Executive function was assessed using Verbal Fluency, Trail Making (Part B), and Stroop-interference tests. The working memory and attention of patients and controls was investigated using Symbol Digit Modalities Test. Finally, the processing speed was checked using Stroop-Word Reading and Stroop-Colour Naming tests.

To evaluate the potential association between use of metformin and ameliorated cognitive function among HD patients, the optimal analysis would have consisted in comparing HD individuals taking metformin against HD individuals not taking metformin. Unfortunately, this comparison was not feasible with the Enroll-HD database since both groups were not comparable as metformin users were all diabetic and diabetes is known to negatively affect cognitive test results (reviewed by Zilliox and coworkers [18]). A doubled blind placebo controlled clinical trial using metformin, in non-diabetic HD patients, would be required compare HD patients taking metformin vs not taking.

Thus, we designed the reasonable alternative to analyze the data within the Enroll-HD cohort: assuming that diabetes affects cognitive performance in a similar way in both HD patients and non-HD individuals then, if metformin had a positive effect in HD patients, the effects of metformin intake would be different between HD patients and non-HD individuals. More specifically, we would expect that metformin intake would associate with lower cognitive performance in the case of non-HD individuals, because they are diabetic. Conversely, metformin intake would associate with higher or at least not lower cognitive performance in the case of HD individuals.

**Fig 1:**
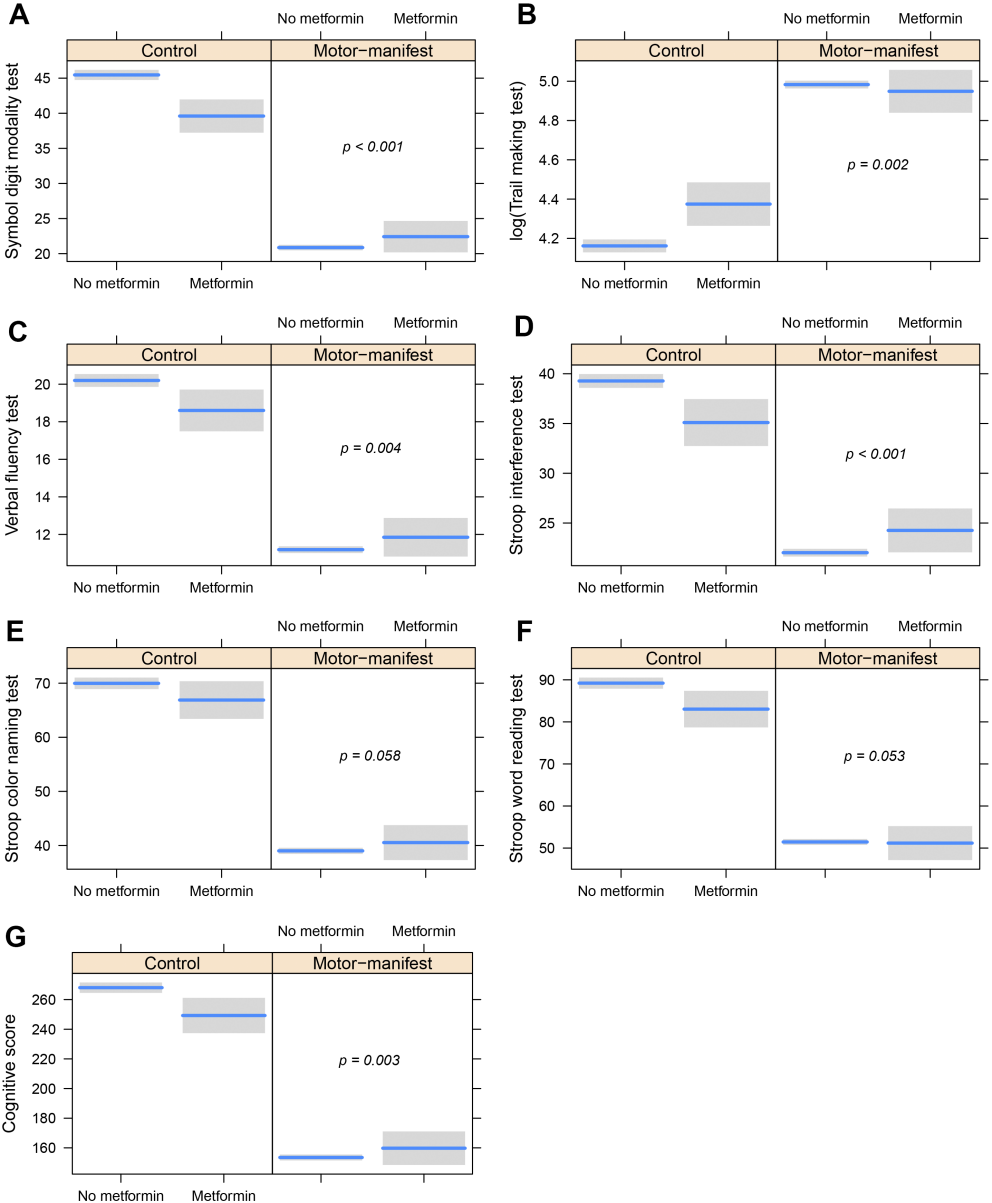
Partial dependence plots showing the interaction between metformin intake and HD status regarding cognitive scores. (A-F) Result of the analysis of the different cognitive tests. G Graph showing the result of the analysis of all cognitive tests (Cognitive Score). These plots are produced using the estimates from the fitted linear regression models, so cognitive values are adjusted for age, gender, BMI and ISCED. The p-values included are assessing the effect of the interactions, that is, the differential effect of metformin intake in HD-patients compared to controls.

Hence we have analyzed the differences between HD motor manifest patients that take metformin versus similar patients that do not. We choose to analyze only HD motor manifest patients, to avoid including participants that carry the mutation, that are far from their age at onset. The results of the different linear models for each cognitive test and the UHDRS cognitive score are as follows: 1) a statistically significant interaction between metformin use and status of HD was found in the case of Verbal fluency (p = 0.004), Stroop interference (p < 0.001), Symbol Digit Modalities (p < 0.001) and Trail Making (p = 0.002) tests; 2) the Stroop word reading (p = 0.053) and the Stroop color naming tests (p = 0.058), showed a similar trend. A representation of the results regarding the adjusted interactions for each model is provided to ease the understanding of the linear modeling results (Fig 1).

### HD patients that take metformin do not show statistically different UHDRS Motorscore

**Fig 2:**
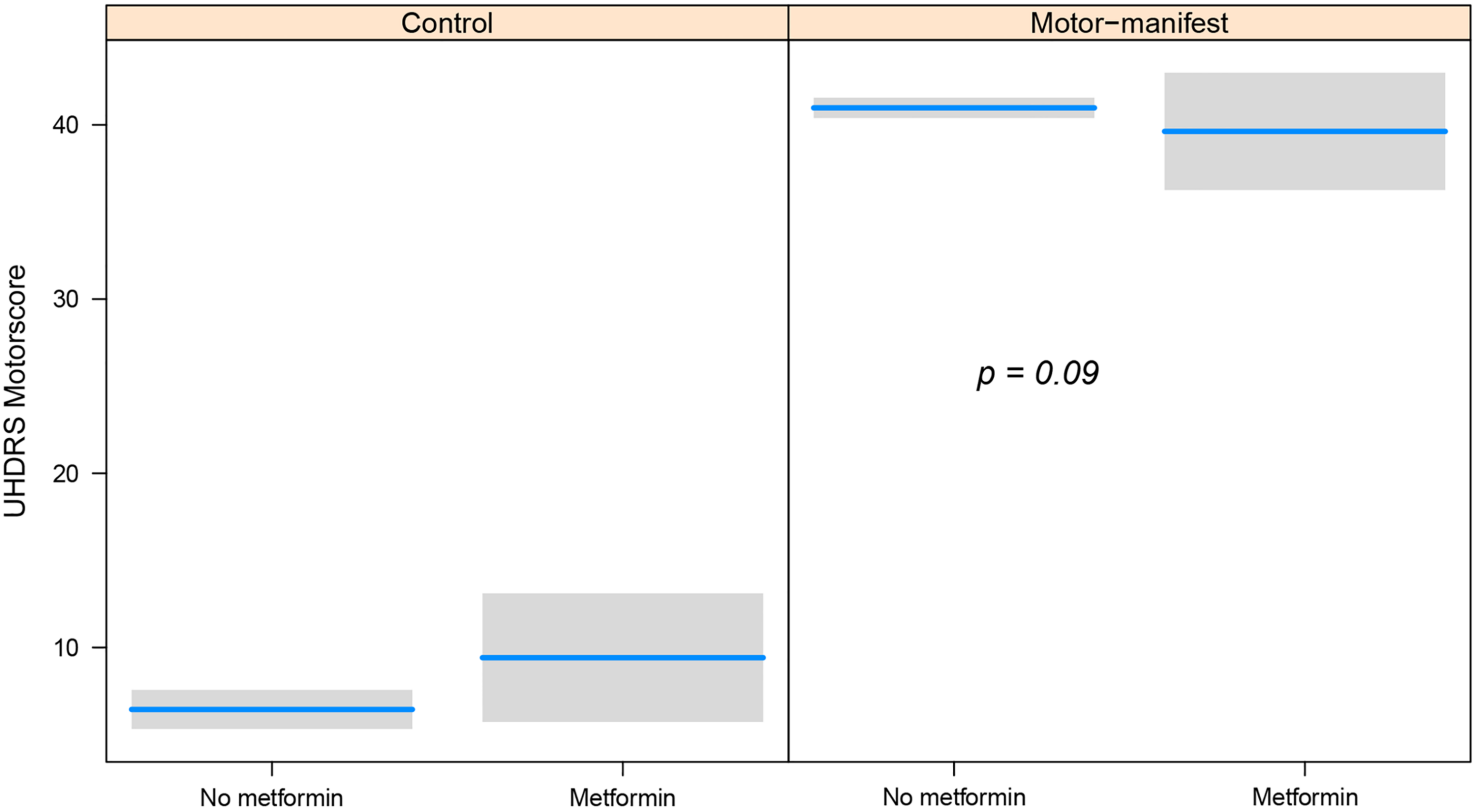
Partial dependence plot showing the interaction between metformin intake and motor impairment (UHDRS Motorscore). Result of the analysis of the UHDRS Motorscore. This plot was produced using the estimates from the fitted linear regression models, so the UHDRS Motorscore values are adjusted for age, gender, BMI and ISCED. The p-value is assessing the effect of the interaction, that is, the differential effect of metformin intake in HD-patients compared to controls.

Since metformin intake showed such strong interaction with better cognitive function in HD patients, we asked ourselves whether this would be observed as well in their motor function. Therefore, we analyzed UHDRS Motorscore using the same linear models used with the cognitive score. Although the analysis showed a trend for metformin takers to be better, this difference was not statistically significant (p = 0.09) (Fig 2).

## Discussion

In this study we performed a statistical analysis of the Enroll-HD cohort (December 2016 release) to assess the relationship between metformin use and cognitive status in HD patients and healthy controls. We have controlled multiple variables such as educational status (ISCED), BMI and age that could potentially contribute to different patterns of cognitive performance. As all the participants that take metformin do so to treat their type 2 diabetes, our statistical approach was based on assessing the interaction between metformin intake and HD status using linear models.

Our novel analysis shows that in HD patients the use of metformin is associated with better results on cognitive tests. For our analysis we took advantage of the fact that in both populations (patients and controls) there is a proportion of individuals who are being treated for type 2 diabetes using metformin (2.8% and 7.4% respectively). Due to the small number of people taking metformin represented in the Enroll-HD database it is not yet possible to analyze the association of metformin over a period of time on the health of HD patients. Despite this limitation, our analysis indicates that metformin intake clearly associates with better cognitive function of these patients.

In contrast with the cognitive status of HD patients that take metformin, the motor function didn’t show a statistically significant improvement, though it showed a trend (Fig 2). In this regard, it is tempting to speculate that HD patients have better neuronal function and this reflects in several phenotypes, not only in their cognitive function. However, we cannot rule out that metformin may have just an effect on the symptoms, rather than improving neuronal function. Why this effect is not statistically significant can be explained by the nature of the motor tests, which methods of data collection are in general more complex and prone to different interpretations, and therefore are less sensitive to change, than cognitive tests.

Metformin has for many years been the first-line drug for the treatment of diabetes type 2 [19]. However, it has also proved useful in the treatment of other diseases, such as polycystic ovary syndrome [16], cardiovascular disease and cancer (see for example [20,21,22]). Despite research to clarify the mechanisms by which this drug acts at the cellular level our understanding of the basis for its effectiveness in a variety of etiologies is still poor. One simple and plausible explanation is that this substance has a pleiotropic effect on tissues and cells. For example, it has been described that metformin sensitizes cells to respond more efficiently to insulin [23], although it is also able to activate glucose transporters to facilitate glucose intake by cells [24], among other potential effects.

At the cellular level several targets of metformin have been described (reviewed by Viollet and co-workers [25]). For example, it has been shown that metformin is a mild inhibitor of the complex I of the electron transfer chain, in mitochondria [26]. This leads to a lower ATP concentration and higher AMP levels, which in turn results in activation of AMP-activated protein kinase (AMPK), activating pro-health span events such as autophagy, among others. In this regard, it has been shown that metformin protects dopaminergic neurons in mouse models of Parkinson disease, through a AMPK-dependent activation of autophagy [27]. Moreover, some studies have shown that activation of AMPK, by metformin or by other means, alleviates phenotypes related to HD in *in vitro* and *in vivo* models of this disease [8,9]. These results are in agreement with the results presented in the current article and strongly suggest that metformin may be used to treat HD symptoms.

It has been shown that type 2 diabetes mellitus has a negative impact on the cognitive function of patients (reviewed by Zilliox and coworkers [18]). The results of our study are compatible with this conclusion as the presence of type 2 diabetes in controls and pre-manifest carriers of the mutation is associated with a trend to lower marks in cognitive tests (Fig 1). However, metformin treated HD patients that also have type 2 diabetes show statistically significant better scores in some cognitive tests (Symbol Digit Modality Test, Verbal Fluency Test, Stroop Interference Test and Trail Making Test) and a trend to higher marks in the rest. How can these results be reconciled? One explanation may be that cognition in HD patients that are also type 2 diabetics, is more severely affected (and by different mechanisms) than in non-HD type 2 diabetics. Hence, metformin in HD patients is able to function in a range of pathways (e.g. on glycaemia to alleviate type 2 diabetes, on autophagy to clear out mutant huntingtin, etc.) to improve cognitive function. We cannot rule out the possibility that type 2 diabetes is improving cognitive function in HD patients, and that this is what we observe in our analysis. However, we believe that is reasonable to say that this seems improbable. Moreover, HD patients may have other conditions, and/or drugs against these conditions, that may alter their cognitive status. These potential confounding factors cannot be taken into account in our study, due to the number of participants taking metformin.

Another potential confusing variable is the weight of patients, which is widely believed to affect the progression of the disease in HD patients [28,29,30] and in rodent models of HD [31]. Hence, it is widely believed that weight loss has a very negative impact in the progression of the disease. However, all analyses described in the present work included BMI as control variable, so our results already correct for differences in BMI values between groups. That means that the effect of metformin is estimated by comparing groups were the differences in BMI have been nullified (i.e. they have the same BMI).

Based on the results of this observational study, together with previous preclinical studies [8,9] we believe that it is important to undertake further basic studies to gain mechanistic insights about the effect of metformin, in HD models. And more importantly, these results indicate the need of clinical trials with metformin to investigate its potential beneficial effects on non-diabetic HD patients.

## Methods

### Sample

We analyzed in this study controls and HD manifest patients, included in the Enroll-HD database as of December 2016 (https://www.enroll-hd.org), whose entry included pharmacologic treatment (n = 7000). Enroll-HD is a global clinical research platform designed to facilitate clinical research in Huntington’s disease. Core data sets are collected annually on all research participants as part of this multi-center longitudinal observational study of Huntington’s disease. Data are monitored for quality and accuracy using a risk-based monitoring approach. All sites are required to obtain and maintain local Ethics Committee approvals. More information can be found at https://clinicaltrials.gov/ct2/show/NCT01574053.

### Clinical and functional assessments

The Unified Huntington’s Disease Rating Scale—total motor score (UHDRS-TMS) [32] was used to assess the presence of a wide range of motor alterations characteristic of HD. These include oculomotor function, dysarthria, chorea, dystonia, Parkinsonism, postural instability, and gait. The UHDRS-TMS is the sum of all individual item scores, with higher scores indicating greater impairment.

Cognitive performance was assessed using the UHDRS cognitive score [32]. The Cognitive score consists of the sum of the scores of five cognitive tasks: the Verbal Fluency Test (FAS), the Stroop word naming, the Stroop color naming, the Stroop interference, and the Symbol Digit Modality Test (SDMT). We also recorded the performance on parts A and B of the Trail Making Test. As a rule, higher score in these tests indicate better performance, since it indicates more hits achieved by the individual. In the trail making test, in contrast, lower marks indicates less time to finish the test, and hence better cognitive function.

### Statistical analyses

Continuous variables were summarized using mean (standard deviation) and median (1^st^, 3^rd^ quartiles). Categorical variables were summarized using absolute and relative frequencies (%). To assess the association between metformin intake and cognitive function seven different linear models were fitted, each one including the score of a different cognitive test as response variable. These linear models included as predictors metformin intake and HD status. Age, body mass index and educational status, based on ISCED (International Standard Classification of Education; UNESCO Institute for Statistics), were also included as covariates to control for them and avoid confounding and effect modification. Since all patients with metformin intake were diabetic and diabetes is known to negatively affect cognitive test results, we also included an interaction between metformin use and HD status to avoid this bias. In the case of the Trail Making Test a logarithm transformation was applied to the values to avoid heteroscedasticity. P values < 0.05 were considered statistically significant. All statistical analyses were performed using R (version 3.3.2).
